# ATP and ACh Evoked Calcium Transients in the Neonatal Mouse Cochlear and Vestibular Sensory Epithelia

**DOI:** 10.3389/fnins.2021.710076

**Published:** 2021-09-08

**Authors:** Richard D. Rabbitt, Holly A. Holman

**Affiliations:** ^1^Department of Biomedical Engineering, University of Utah, Salt Lake City, UT, United States; ^2^Graduate Program in Neuroscience, University of Utah, Salt Lake City, UT, United States; ^3^Department of Otolaryngology-Head and Neck Surgery, University of Utah, Salt Lake City, UT, United States

**Keywords:** development, supporting cell, hair cell, purinergic, cholinergic, sensory cell

## Abstract

Hair cells in the mammalian inner ear sensory epithelia are surrounded by supporting cells which are essential for function of cochlear and vestibular systems. In mice, support cells exhibit spontaneous intracellular Ca^2+^ transients in both auditory and vestibular organs during the first postnatal week before the onset of hearing. We recorded long lasting (>200 ms) Ca^2+^ transients in cochlear and vestibular support cells in neonatal mice using the genetic calcium indicator GCaMP5. Both cochlear and vestibular support cells exhibited spontaneous intracellular Ca^2+^ transients (GCaMP5 ΔF/F), in some cases propagating as waves from the apical (endolymph facing) to the basolateral surface with a speed of ∼25 μm per second, consistent with inositol trisphosphate dependent calcium induced calcium release (CICR). Acetylcholine evoked Ca^2+^ transients were observed in both inner border cells in the cochlea and vestibular support cells, with a larger change in GCaMP5 fluorescence in the vestibular support cells. Adenosine triphosphate evoked robust Ca^2+^ transients predominantly in the cochlear support cells that included Hensen’s cells, Deiters’ cells, inner hair cells, inner phalangeal cells and inner border cells. A Ca^2+^ event initiated in one inner border cells propagated in some instances longitudinally to neighboring inner border cells with an intercellular speed of ∼2 μm per second, and decayed after propagating along ∼3 cells. Similar intercellular propagation was not observed in the radial direction from inner border cell to inner sulcus cells, and was not observed between adjacent vestibular support cells.

## Introduction

Functional hearing and balance rely on mature sensory hair cells and precise organization of support cell networks. These sensory hair cells and non-sensory supporting cells coordinate signaling that starts in development and continues throughout adulthood. The release of calcium (Ca^2+^) from internal stores is critical for maturation and activity. Ca^2+^ transients appear to occur spontaneously at rest and can also be evoked by the activation of purinergic and cholinergic receptor binding in different cell populations throughout development. ACh and/or ATP can trigger inositol triphosphate (IP_3_) dependent calcium induced calcium release (CICR) in inner ear non-sensory support cells. Previous studies have shown in the cochlea that binding of extracellular ATP to G-protein coupled P2Y2 and P2Y4 receptors, expressed on the endolymphatic surface of the developing sensory epithelium, activates phospholipase-C dependent generation of IP_3_ (Gale et al., 2004; Beltramello et al., 2005; Piazza et al., 2007; Ceriani et al., 2019). Additionally, muscarinic ACh receptors in the vestibular epithelium triggers IP_3_ dependent CICR in specific vestibular hair cells (Holman et al., 2019, 2020). In some cases, gap junctions between adjacent support cells can lead to relatively slow propagation of intercellular Ca^2+^ waves. In addition, the probability of connexin hemichannel opening is a function of cytosolic free Ca^2+^ peaking at ∼500 nM (De Vuyst et al., 2006) which facilitates the propagation of regenerative and coordinated intercellular Ca^2+^ waves, with peak amplitude of ∼500 nM, sustained by ATP-induced ATP-release (Gale et al., 2004; Beltramello et al., 2005; Mammano et al., 2007; Piazza et al., 2007; Anselmi et al., 2008; Majumder et al., 2010).

In mouse non-sensory cells of the lesser epithelial ridge (LER), purinergic Ca^2+^ transients from intracellular Ca^2+^ wave propagation is sustained by extracellular ATP (Anselmi et al., 2008). In non-sensory cells of the greater epithelial ridge (GER), ATP evokes Ca^2+^ transients with rhythmic bursts (Hinojosa, 1977; Kamiya et al., 2001; for a list of abbreviations see Table 1). The frequency of spontaneous Ca^2+^ transients is reduced by purinergic antagonists. The propagation of intracellular Ca^2+^ waves in the LER and the frequency of spontaneous Ca^2+^ transients in the GER increase with decreasing the extracellular Ca^2+^ concentration (Tritsch et al., 2007; Anselmi et al., 2008; Tritsch et al., 2010), which increases the open probability of connexin hemichannels (Müller et al., 2002; Gómez-Hernández et al., 2003; Sáez et al., 2005; González et al., 2007). Intracellular Ca^2+^ modulation has been suggested to play an important role in regulation of differentiation, cell fate and circuit formation in the cochlea, crista and macula. The present report is focused on comparing and contrasting relatively slow Ca^2+^ transients in the mouse cochlear and vestibular sensory epithelium during the first postnatal week.

**TABLE 1:** List of abbreviations and/or acronyms.

Term cell type	Definition
CSC	Cochlear support cell
VSC	Vestibular support cell
IBC	Inner border cell
HC	Hensen’s cell
DC	Deiters’ cell
ISC	Inner sulcus cell
IPhC	Inner phalangeal cell
IHC	Inner hair cell
OHC	Outer hair cell
LER	Lesser epithelial ridge
GER	Greater epithelial ridge
Clino2 (C2)	Clino2 cell
CT	Clinocyte
VSC-Type A	Vestibular support cell type A
VSC-Type B	Vestibular support cell type B
**Physiology**	
Ca^2+^	Calcium
CICR	Calcium induced calcium release
GCaMP5	Genetically encoded calcium indicator 5
ACh	Acetylcholine
ATP	Adenosine triphosphate
IP_3_	Inositol trisphosphate
ABR	Auditory brainstem response
DPOAE	Distortion product otoacoustic emissions
**Calcium imaging**	
ΔF/F	Change in fluorescence divided by baseline fluorescence
AU	Arbitrary unit
ROI	Region of interest
**Gene/Protein**	
Gad2/GAD2	Glutamate decarboxylase

*Statistical significance of differences in GCaMP5G ΔF/F means reported in Figure 5.*

In the present study the genetic calcium indicator GCaMP5G was used to record Ca^2+^ transients in non-sensory and sensory cells in the organ of Corti, crista ampullaris and utricular macula. We observed spontaneous and evoked Ca^2+^ transients in semi-intact organs during the first postnatal week. The GCaMP5G indicator is suitable for events lasting > 200 ms including G protein dependent CICR but is not capable of tracking fast Ca^2+^ events. We report diverse Ca^2+^ transients evoked by cholinergic and purinergic puff application in non-sensory Hensen’s and Deiters’ cells in the LER and inner border cells (IBCs) and inner phalangeal cells (IPhCs) in the GER. Sensory inner hair cell (IHCs) also responded to ACh and ATP with Ca^2+^ transients during the first week. ATP and ACh evoked Ca^2+^ bursts in Hensen’s cells were similar in range to the ΔF/F rates observed in the eminentia cruciata supporting cells, clino2 and clinocytes and the clino2 cells are a population of progenitor-like cells first identified by immunomorphological characteristics and location in the eminentia cruciata of anterior and posterior canals (Holman et al., 2020). This study demonstrates the robust signaling these and other supporting cells have in the vestibular epithelia along with unique Ca^2+^ transients in multiple supporting cell populations in the cochlear epithelia of the same animal throughout different stages of postnatal development in the mouse.

## Materials and Methods

### Bioethics Statement

Experiments were conducted in accordance with the NIH Guide for the Care and Use of Laboratory Animals and the National Research Council (US) Committee. All mouse procedures were in accordance with animal welfare protocols approved by the University of Utah’s Institutional Animal Care and Use Committee.

### Genotyping of Transgenic Mice

Transgenic mice expressing GCaMP5G on the C57BL/6 background strain were utilized for calcium imaging [*Polr2a^*Tn(pb*–*CAG*–*GCaMP*5g,–*tdTomato*)Tvrd^*; Stock No: 024477] and crossed with mice homozygous for Gad2-Cre [*Gad2^*tm*2(*cre)Zjh/J*^*; Stock No: 010802; this strain is also known as PC-G5-tdT]. Gad2-Cre drives expression in hair cells and supporting cells, thereby allowing simultaneous observation of calcium transients in multiple cell types. Homozygous transgenic mice obtained from The Jackson Laboratory were crossed to generate Gad2^+/GCaMP5G^ heterozygous first-generation transgenic mice used in this study. Gad2-Cre; PC-G5-tdT mice were genotyped by standard RT-PCR using primers specific to GCaMP5G and Cre (Transnetyx). Seven Gad2-Cre; PC-G5-tdT mice aged P1-P6 provided physiology data. Male Gad2-Cre; PC-G5-tdT mice aged P44 (*n* = 5) and P94 (*n* = 5) provided auditory brain stem response (ABR) and distortion product otoacoustic emissions (DPOAE) data.

### Auditory Brainstem Responses

ABRs were conducted on Gad2-Cre; PC-G5-tdT heterozygous mice F_1_ generation from two age groups to test auditory function. In brief, mice were anesthetized by intraperitoneal injection with ketamine (100 mg/kg body weight) and xylazine (10 mg/kg). For adults and older mice, a small incision was made at the tragus to allow direct access to the ear canal. Body temperature was maintained at ∼37°C via a heating lamp. ABR and DPOAE recordings were made in a double-walled sound chamber (IAC, Bronx, NY). Evoked potentials were measured by placing needle electrodes over the pinna and vertex in a vertex/mastoid configuration. A ground electrode was inserted subcutaneously near the tail. ABR thresholds were obtained to tone pips at 8, 12, 16, 22, 32, 4 kHz. Stimuli were presented over a 15–90 dB SPL range of intensity in 5 or 10 dB steps. ABR signals were amplified (TDT RA4), filtered 100 Hz to 3 Hz, and averaged (1024 sweeps; TDT RA16BA controlled by BioSigRP software; Tucker-Davis Technology). Threshold responses were determined by visual inspection of ABR waveforms. The cochlea was considered to be non-responsive if no signal was recorded at 90 dB SPL. Auditory phenotypes in two age groups young (P44; ∼1 month) and adult (P94; ∼3 months) male Gad2-Cre; PC-G5-tdT transgenic mice were tested.

### Distortion Product Otoacoustic Emissions

Otoacoustic emission (OAEs) were measured using a microphone coupled with two speakers (ER-10B+ and 2xEC1 Etymotic Research, Elk Grove, IL). Stimuli of two primary tones, *f*_1_ and *f*_2_, with *f*_2_/*f*_1_ = 1.2 and *f*_2_ level 10 dB < *f*_1_ level were recorded. Primary tones were stepped from 30 to 80 dB SPL (for *f*_1_) in 10 dB increments and swept in octave steps at 8, 22, 32 kHz. The ear canal acoustic emissions were amplified and digitized. Signals at *f*_1_, *f*_2_, 2*f*_1_–*f*_2_, were determined by FFT after spectral averaging from 50 waveform traces, each corresponding to 84 ms of digitized ear canal sound pressure waveform. Statistical analysis of ABR and DPOAE data was performed, and data are shown in standard error of the mean ± SEM.

### Tissue Preparation

Semi-intact vestibular preparations or apical and mid-turn sections of the cochlea from Gad2-Cre; PC-G5-tdT mice of either sex were studied from acutely dissected bony labyrinths in 4°C extracellular buffer [EB (mM): 5.8 KCl, 155 NaCl, 0.9 MgCl_2_, 1.3 CaCl_2_, 0.7 NaHPO_4_, 5,6 glucose, 10 HEPES, 1 Na pyruvate, pH 7.4; osmolality ∼308 mmol kg^–1^] (Holman et al., 2019). Dissected tissue from postnatal days 4–7 (P4-P7; day of birth is P0) were transferred to a recording chamber and immobilized with a nylon harp (Warner Instrument, RC-22C), and continuously perfused with EB at room temperature (21–23°C). Live cell calcium imaging was recorded up to a maximum 4 h post dissection.

### Calcium Imaging

Swept field confocal microscopy (Bruker, United States) was used for live cell calcium recordings. Fluorescent confocal images were formed using water immersion objective 40X/N.A.0.8 or 20X/0.5W (Olympus, Tokyo, Japan) controlled by Prairie View (Bruker). Confocal images were collected using a 35 μm slit aperture in linear galvanometer mode, and a 512 × 512 detector (Photometrics, RoleraMGi Plus EMCCD) with in-plane single pixel size for the 40 × objective of 0.27 × 0.27 μm. For GCaMP5G fluorescence excitation was limited to a 488 nm diode laser and the detection filter was a band pass filter (525/50–25, Semrock/IDEX Health and Science, LLC, Rochester, NY). For each record, 1,000–2,000 GCaMP5G fluorescence images were captured at 5 frames-s^–1^. In a subset of recordings, ACh or ATP were applied 10 s into the record for a duration of 0.1–5 s using a pressure driven micro-manifold with a ∼100 μm tip located ∼1 mm from the tissue (ALA Scientific, QMM). Concentration in the manifold was 100 μM leading to < 100 μM (∼50 μM) at the location of the tissue. Each xy image was smoothed in space with a 3 pixel Gaussian filter (WaveMetrics, Igor). To minimize motion artifact, images were registered in space (Holman et al., 2019) over time using manually selected regions of interest. GCaMP5G fluorescence modulation was determined pixel-by-pixel using ΔF/F_*MIN*_, (or, ΔF/F) where ΔF = F(t)−F_*MIN*_ and F_*MIN*_ was the minimum fluorescence intensity in the pixel over the entire time sequence of images. GCaMP5G has a K_*D*_ of 0.41 μM and a decay time of t_1/2_ 154 ms (Sun et al., 2013).

### ΔF/F Transients and Statistical Analysis

Unless otherwise noted, ΔF/F was determined as a function of time within 10 μm diameter regions of interest (ROI) identified with specific cells. Multiple ROIs were analyzed for each image sequence as indicated in individual figures. ΔF/F curves reported in the figures show the peak *f(t) = max(ΔF/F)* within each ROI as functions of time “t” (or frame number). The onset time of a Ca^2+^ transient relative to the stimulus was determined by the time when *f(t)* emerged above the average noise in the ROI. Each transient was analyzed to find the onset time, peak ΔF/F, rise time, half width and fall time. For evoked transients, the latency was defined as the onset time of the first transient minus the onset time of the stimulus. If multiple repetitive transients occurred in a single ROI the average time between onsets was determined and reported as the period (or rate = 1/period). Mean values were determined for each statistic (peak, rise, half, fall, rate), grouped by cell type (e.g., Hensen’s, Deiters’…). Error bars denote ± SEM (standard error of the mean). Transients in different cell types were compared pairwise using Student’s *t*-test. *i*-values (P) where < 0.05 was the criterion for statistical significance. The experiments were not blinded during the experiment or analysis.

### Data Availability

Data generated and analyzed for this study are included in this publication. Datasets generated during the study are available from the corresponding author upon request.

## Results

### Spontaneous and ATP Evoked Ca^2+^ Transients in Cochlear GER and LER Cells

**FIGURE 1:**
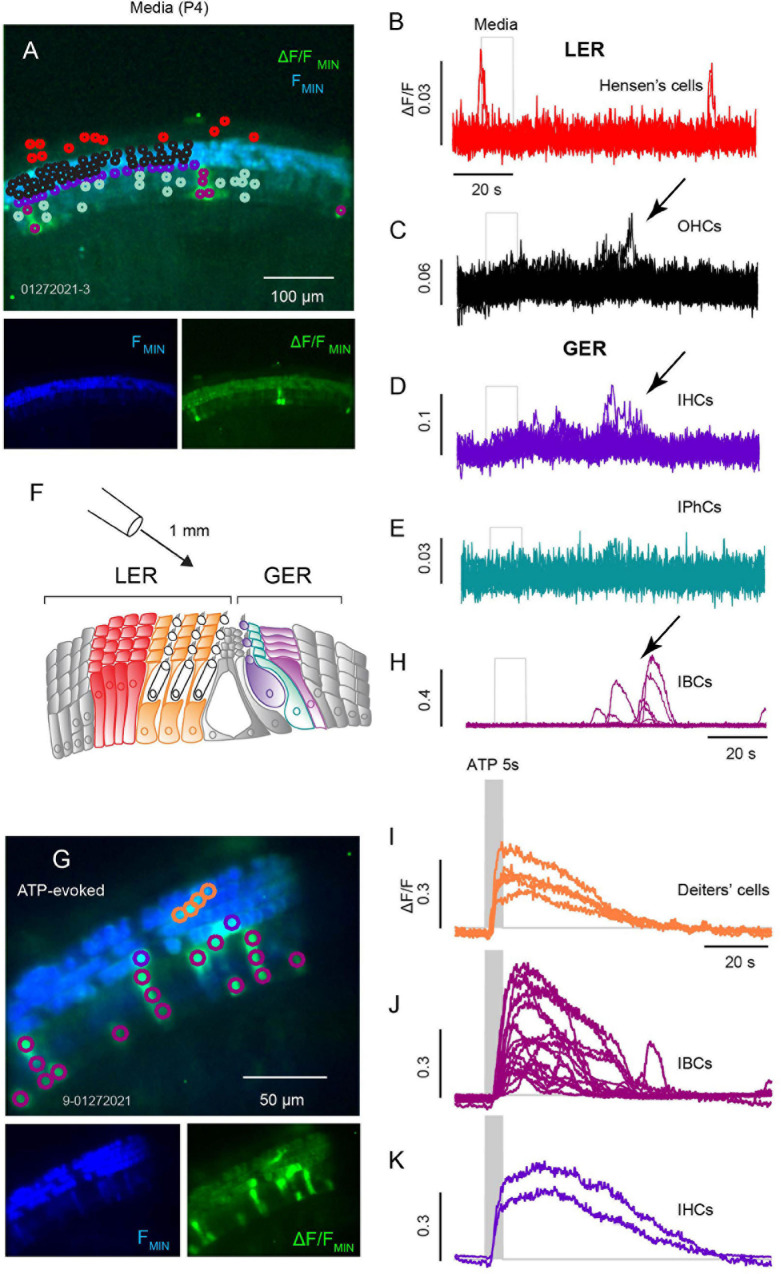
Spontaneous and ATP evoked Ca^2+^ transients across sensory and non-sensory cells in the organ of Corti. **(A)** Confocal image of spontaneous Ca^2+^ transients in an apical turn with baseline GCaMP5G fluorescence (blue) overlaid with ΔF/F_*MIN*_ (green) with Ca^2+^ transients during a 100 s recording. Traces from ROIs overlying Hensen’s cells **(B)**, OHCs **(C)**, IHCs **(D)**, IPhCs **(E)**, and IBCs **(F)**. **(G)** Illustration of a cochlear section with Hensen’s cells (red) and Deiters’ cells (orange) of the lesser epithelial ridge (LER), IHCs (purple), IPhCs (cyan), and IBCs (fuchsia) in the greater epithelial ridge (GER). (H) Image with a 40x obj. following a 10 s ATP (100 μM) puff positioned ∼1 mm from the semi-intact cochlea. Ca^2+^ transients in Deiters’ cells, IHCs, and IBCs. **(H,I)** Traces from ROIs overlying Deiters’ cells with ATP evoked Ca^2+^ transient **(I)**; traces from ROIs overlying IBCs **(J)**; and traces from ROIs overlying IHCs **(K)** (see Supplementary Video 1).

During development Ca^2+^ activity in the sensory epithelia of the cochlea, semicircular canals and otolith organs facilitate mapping in the central nervous system laying the foundation for auditory and vestibular function. To measure this activity, we recorded Ca^2+^ transients in ROIs overlying cells of the GER and lower epithelial ridge (LER) from *ex vivo* cochlear preparations of apical and mid turn sections. Spontaneous ΔF/F transients for ROIs outlined in Figures 1A–H for tissue from a P4 mouse. Colors in Figures 1A–H denote ROIs overlying specific cell types in the LER and GER (Figure 1B, Hensen’s: red, *n* = 12; Figure 1C, OHCs: black, *n* = 46; Figure 1D, IHCs: purple, *n* = 20; Figure 1, IPhCs: cyan, *n* = 17; Figure 1F, IBCs: magenta, *n* = 7). IBC ROIs (Figure 1F) exhibited the most intense spontaneous transients with ΔF/F > 0.5 and duration often lasting longer than 10 s, while IPhCs did not exhibit detectable transients above the noise in this P4 mouse. To note, small transients in ROIs overlying IHCs and OHCs corresponded in time with large spontaneous transients in IBCs (arrows: Figures 1C,D,F), suggesting these events are causally related. It is difficult to completely isolate a single cell using a 20x water immersion objective, and it is likely that small signals in ROIs overlying IHCs (Figure 1D) and OHCs (Figure 1C) was fluorescence from adjacent Deiters’ cells or IBCs (note temporal correspondence in Figures 1C,D,F: arrows). Consistent with this interpretation, large Ca^2+^ transients can be evoked in IHCs and in Deiters’ cells at this age, but not in OHCs. In a subset of records, Ca^2+^ transients in IBCs appeared sequentially in adjacent cells with an apparent intercellular propagation speed of approximately 2 μm-s^–1^ and subsiding after a distance of 1–3 cells. Hensen’s cells also exhibit spontaneous Ca^2+^ activity but occurring at times uncoordinated with transients in other support cells. In this same P4 tissue, 100 μM ATP evoked large Ca^2+^ transients in IHCs, IBCs and Deiters’ cells. Transients were synchronized to the onset of the ATP puff as shown in Figures 1G–K. IBCs responded to ATP with the highest ΔF/F intensity, with Ca^2+^ transients initiating at the apical end of the cells, propagating with an intracellular speed of ∼25 μm-s^–1^ (also see Supp. Video 1), consistent with typical speed of intracellular CICR waves in other cell types.

### ATP Evoked Ca^2+^ Transients in the Developing Cochlea

**FIGURE 2:**
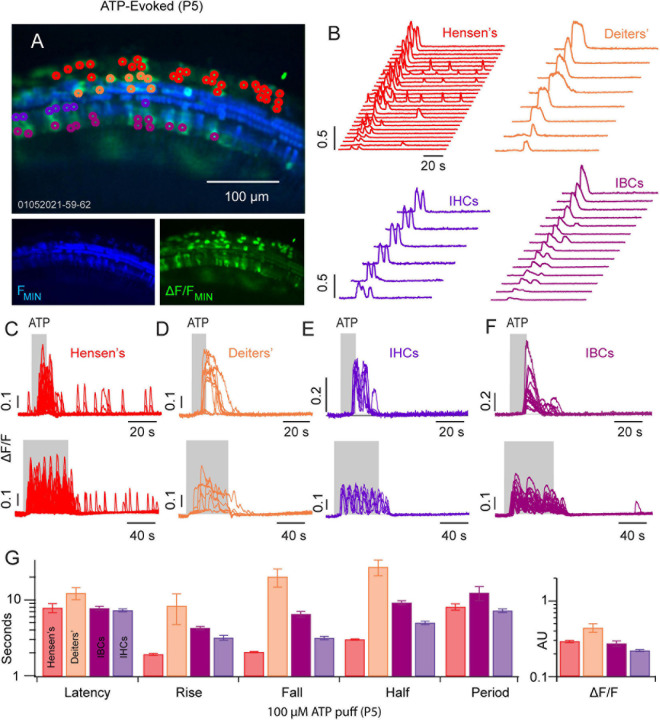
ATP evoked Ca^2+^ transients propagate across the GER and LER. **(A)** Image of ATP evoked Ca^2+^ transients in a P5 cochlea mid-turn region. **(B)** Waterfall plots with traces from ROIs overlying Hensen’s cells (red), Deiters’ cells (orange), IHCs (purple) and IBCs (fuchsia). **(C–F)** Illustrate the timing of evoked transients for a 10 s (top panels) and for 50 s ATP (100 μM) puff application. **(G)** Statistics showing latency to first evoked transient, rise time, fall time, half width, period, and peak ΔF/F in each cell type (see Supplementary Video 2).

We next examined ATP evoked Ca^2+^ transients in a P5 mouse following two different ATP exposures: 10 and 50 s puff of ATP (100 μM). Hensen’s cells (Figures 2A–C: red ROIs and traces, *n* = 32) responded to a 10 s ATP puff with a short burst of 2–4 Ca^2+^ transients (Figures 2A,B; *n* = 32 cells). Prolonged exposure to ATP (50 s) gave an increased number of Ca^2+^ transients within each burst (Figure 2C). Smaller Ca^2+^ transient activity remained in Hensen’s cells following the ATP puff with reduced ΔF/F (∼0.1 AU). ATP evoked Ca^2+^ transients in Deiters’ cells (Figures 2A,B,D: orange, *n* = 8) appeared as a single pulse during the ATP exposure without a clearly discernable bursting. IHCs (Figures 2A,B,E: purple, *n* = 6) and IBCs (Figures 2A,B,F: magenta, *n* = 14) also responded to ATP with a burst of Ca^2+^ transients (Figures 2C–F; see Supplementary Video 1). The onset latency, rise time, fall time, half width, burst period, and ΔF/F magnitude are shown in Figure 2G. Onset latencies were similar in all 4 cell types, but the stereotypical shape and rate of ATP evoked transients differed between cell types. Together, these results suggest that non-sensory and sensory cells spanning the LER and GER have a coordinated Ca^2+^ response to ATP at P5 prior to the maturation of hearing.

### ATP vs. ACh Evoked Ca^2+^ Transients in the Developing Cochlea

**FIGURE 3:**
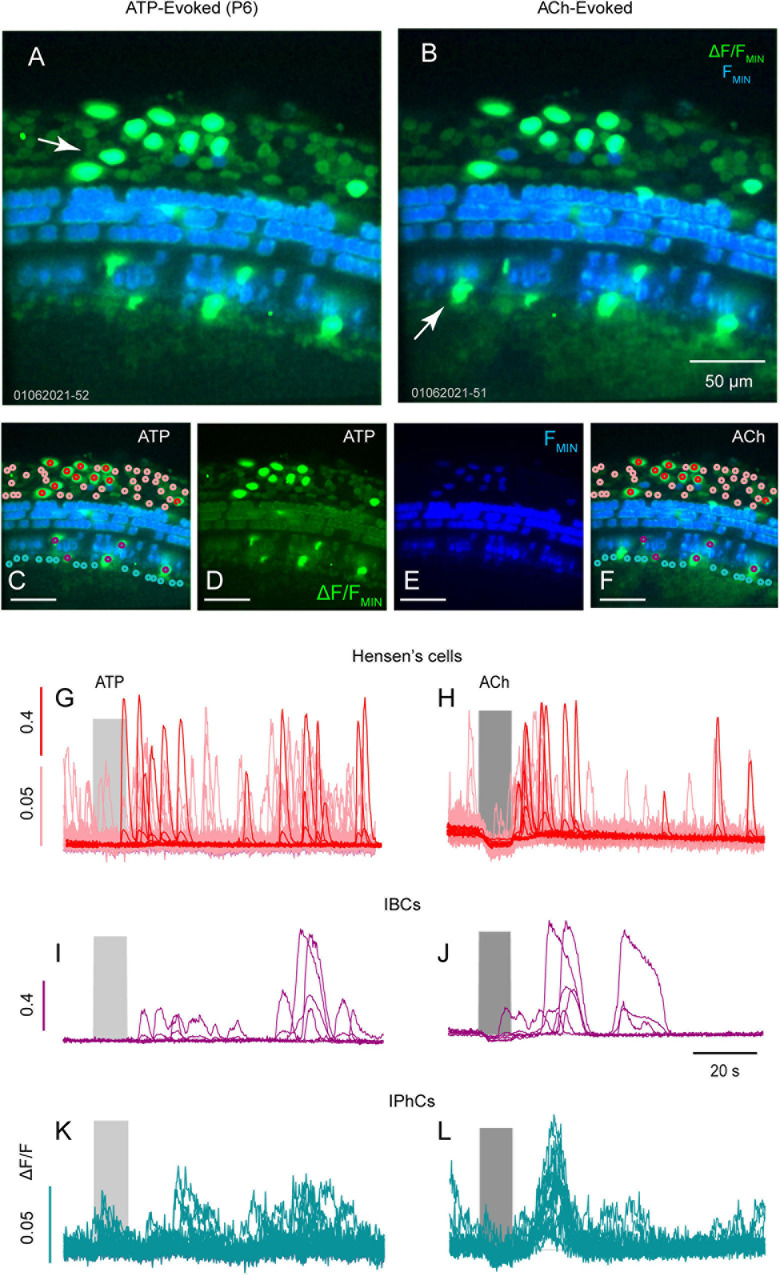
ATP and ACh evoked Ca^2+^ transients in Hensens’s cells, IBCs and IPhCs. Confocal images of ATP **(A,C,D)** and ACh **(B,E,F)** with baseline GCaMP5G fluorescence (blue) overlaid with ΔF/F_*MIN*_ (green) from a mid-turn cochlea age P6. Maximum ΔF/F_*MIN*_ evoked by ATP and ACh in ROIs overlying Hensen’s cells (**G,H**; red and pink), overlying IBCs (**I,J**; cyan) and overlying IPhCs (**K,L**; fuchsia).

We examined Hensen’s cells, IPhCs and IBCs for Ca^2+^ transients evoked by ATP and ACh in a P6 mouse (Figures 3A–C). At P6, Hensen’s cells (Figures 3A–H: red and pink), responded with Ca^2+^ bursts following 100 μM ATP (Figure 3G) and ACh (Figure 3H). Ca^2+^ transients in Hensen’s cells fell into two response groups at P6; cells with large ΔF/F∼1 (Figures 3G,H: red) and cells with small ΔF/F∼0.1 (Figures 3G,H: pink). Small Ca^2+^ transients (pink) were not evoked in Hensen’s cells by ATP or ACh, while large transients (red) were evoked by either compound (Figures 3H,J,L: The baseline depression during ACh application is motion artifact). Evoked Ca^2+^ transients in IBCs (Figures 3A–F,I,J: cyan) were similar in shape for ATP (Figure 3I) and ACh (Figure 3J) but the latency was increased in response to ATP relative to ACh. The delayed ATP Ca^2+^ transients in non-sensory cells reported by others suggest this may involve ATP- and inositol 1,4,5-triphiosphate (IP3) cytosolic free Ca^2+^ oscillations (Ceriani et al., 2019). In IPhCs, ATP did not evoke Ca^2+^ transients above the small spontaneous events, while ACh evoked larger highly synchronized transients (also see Supplementary Video 2). The present report does not examine the origin of differences between ATP vs. ACh evoked responses in these cells.

### ATP and ACh Evoke Ca^2+^ Transients in Supporting Cells of the Crista Ampullaris

**FIGURE 4:**
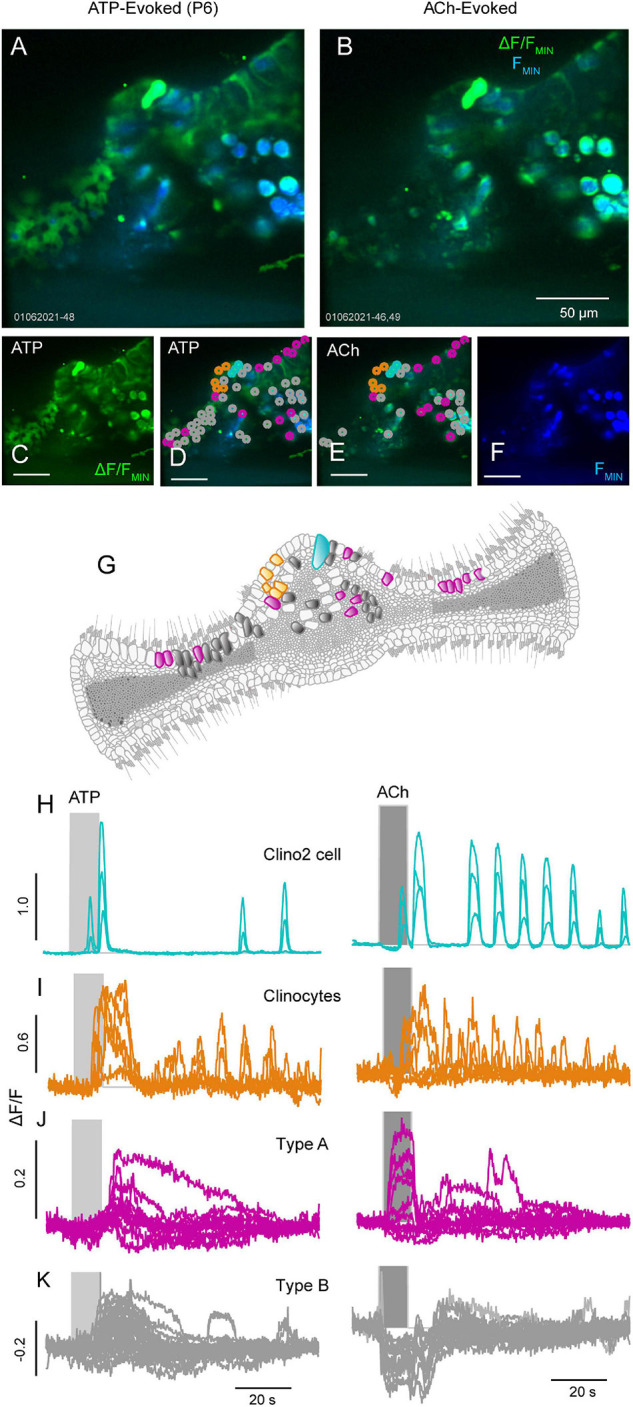
ATP and ACh evoked Ca^2+^ transients in the crista. Confocal images of an anterior canal crista with baseline GCaMP5G fluorescence (blue) overlaid with ΔF/F_*MIN*_ (green) transients evoked by 100 μM ATP **(A,C,D)** or ACh **(B,E,F)** in a P6 mouse. **(G)** Illustration of the anterior canal crista mapping the color-coded supporting and sensory hair cells (not drawn to scale). The clino2 cell (**H**; teal) and clinocytes (**I**; orange) respond to ATP and ACh with evoked Ca^2+^ transients. Two other cell groups denoted type A (fuchsia) and type B (grey) also respond to ATP and ACh with evoked Ca^2+^ transients **(J,K)** but with distinctly different time courses (see Supplementary Videos 3, 4).

Previous studies from our lab have shown supporting cells in the non-sensory region of the crista have spontaneous and acetylcholine (ACh) evoked Ca^2+^ transients during early development (Holman et al., 2019, 2020). Here, we extend those studies to examine purinergic and cholinergic Ca^2+^ signaling in support cells during postnatal day 6 (Figures 4A–G, also see Supplementary Videos 3, 4). In the anterior and posterior semicircular canals, supporting cells in the peripheral zone of the crista and recently identified supporting cells, clino2 and clinocytes (Holman et al., 2020), in the eminentia cruciata (EC) have distinct ATP and ACh evoked Ca^2+^ transients (Figure 4). In clino2 (Figures 4D,E,G,H: cyan), 100 μM ATP (Figure 4H, left) and ACh (Figure 4H, right) both evoke bursts of Ca^2+^ transients. A single one clino2 cell is present in Figure 4, with multiple traces showing different ROIs within a single cell (Figure 4H). Evoked Ca^2+^ transients in the clino2 cell start at the apex facing endolymph and propagate down the cell as a wave consistent with previous studies (Holman et al., 2020). While the rise time for Ca^2+^ transients in the clino2 cell are similar when evoked by 100 μM ATP vs. ACh, the number of Ca^2+^ events in clino2 were larger following ACh for the 100 μM dose. Clinocytes also respond to both ATP and ACh with bursts of Ca^2+^ transients. However, the temporal envelope differed between the two compounds with markedly different Ca^2+^ transients in the clino2 cell and clinocytes. Although the present report did not examine the specific receptors involved, it has been shown previously that ACh responses in clino2 and clinocytes are muscarinic and sensitive to compounds blocking IP_3_-dependent CICR (Holman et al., 2020).

It is difficult to precisely identify specific cell types in the sensory region of the crista from GCaMP5G images, therefore other cell types were divided into two groups (Figures 2J,K) based on Ca^2+^ responses to ACh (Excite: A, Other: B). Both type A (magenta) and type B (gray) cells responded to ATP with small excitatory Ca^2+^ transients following a 10 s puff application (Figures 2J,K), but responses during the puff itself differed between cells. The most notable difference was the short latency ΔF/F during the ACh pulse. Cell type A responded to ACh with a positive ΔF/F during the pulse that returned to zero immediately upon wash, at the maximum speed of the GCaMP5G indicator. Responses to ACh (Figure 4J) are consistent with activation of a nicotinic receptor, but the present study did not distinguish receptors or cell types. Similar responses were not observed in cochlear cells examined in the present study.

### Purinergic Signaling in Support Cells in the Utricular Macula

**FIGURE 5:**
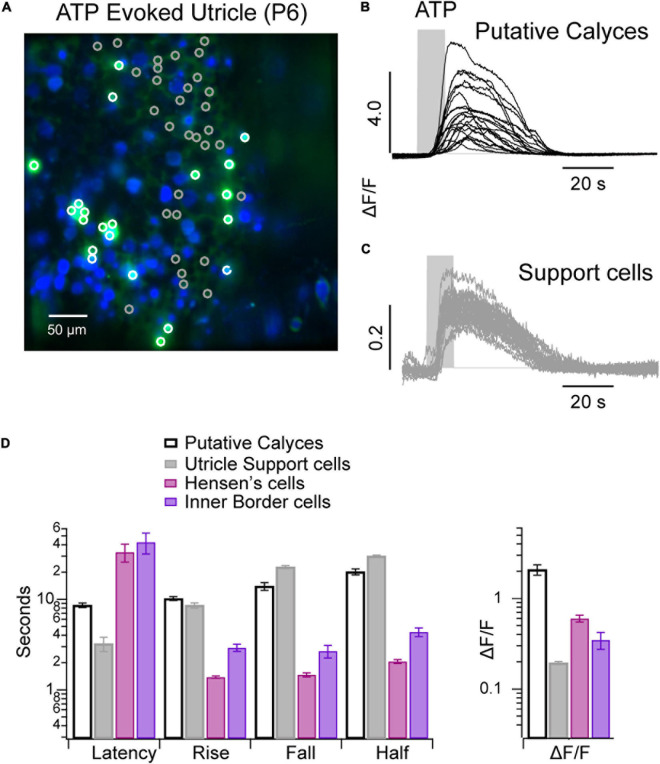
ATP evoked Ca^2+^ transients in the utricular macula. **(A)** Confocal image of a P6 utricular maculae with ATP evoked Ca^2+^ transients ΔF/F_*MIN*_ (green) overlaid with baseline GCaMP5G fluorescence (green). **(B)** Ca^2+^ transients in calyces (white ROIs/black traces). **(C)** Ca^2+^ transients in support cells (gray ROIs/traces). **(D)** Bar chart summarizing ATP evoked Ca^2+^ transients in non-sensory cells from cochlea and utricle of same P6 mouse (see Supplementary Video 5).

Spontaneous and ACh evoked Ca^2+^ transients in utricular hair cells and supporting cells have been reported previously during the first postnatal week, but ATP evoked transients were not observed (Holman et al., 2019, 2020). Here, we report the presence of long-lasting Ca^2+^ transients evoked by 100 μM ATP (Figure 5). A subset of cells responded to ATP with intense (ΔF/F > 5 AU) long lasting (>20 s) Ca^2+^ transients (Figure 5A: white ROIs, Figure 5B: black traces; Figure 5D), while the remaining cells responded over a similar time course with much smaller Ca^2+^ transients (ΔF/F < 0.4). The difference in ΔF/F identifies these as two different cell types. Background fluorescence and morphology implies cells with large ΔF/F transients are likely to be calyces (labeled “putative calyces,” Figure 4B). A subset of utricular hair cells in this transgenic mouse line express high levels of calcium bound GCaMP5, which generate continuous background fluorescence (Figure 5A, blue). Based on morphology, these hair cells are type I (Holman et al., 2020). Cells with large ATP-evoked ΔF/F transients always enveloped a highly expressing cell with type I morphology, suggesting they are likely to be calyces. This feature is most easily seen in the video where the large ΔF/F transients (green) develop around and envelope another cell of smaller diameter (blue) (see Supplementary Video 5). Gray ROIs (Figure 5A) all exhibited small ATP-evoked ΔF/F transients with nearly identical latencies and duration. Based on their spatial pattern and between hair cells these cells are most likely support cells, and are labeled as such in Figure 5C. The onset latencies were significantly shorter in utricular vs. cochlear supporting cells (Figure 5D), while the kinetics were significantly slower in utricular supporting cells in a P6 mouse.

### Kinetics of ACh vs. ATP Evoked Ca^2+^ Transients in Non-sensory Cells in the Organ of Corti and the Anterior Canal Crista

**FIGURE 6:**
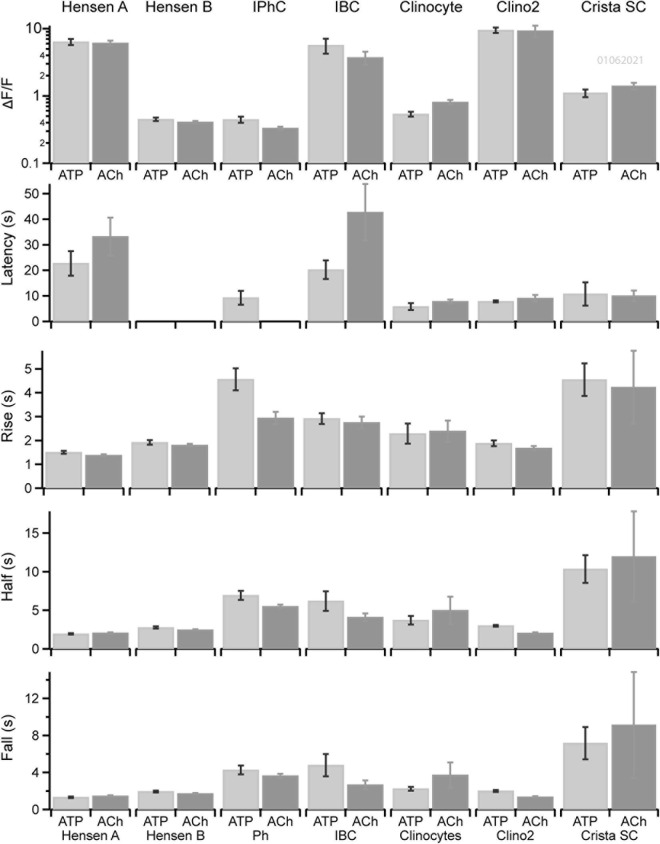
Kinetics of Ca^2+^ transients in non-sensory cells in cochlea and crista. Kinetics of ATP (dark gray) or ACh (light gray) evoked Ca^2+^ transients in ROIs overlying Hensen’s cells (type A), Hensen’s cells (type B), IPhCs, clinocytes, the clino2 cell, and crista supporting cells across multiple cells of the same type in a P6 mouse. Bars show the mean and standard error of peak ΔF/F, latency to the first evoked transient relative to the stimulus onset, rise time of the transient, width at half peak amplitude and fall time.

The kinetics of ATP and ACh evoked GCaMP5G ΔF/F transients differed between support cell types in the cochlea and vestibular sensory epithelium (e.g., Figures 1–5). A direct comparison between Ca^2+^ transients in supporting cell types in the organ of Corti and the crista is provided in Figure 6 for tissue in a single ear during one experimental session. Statistics shown are: Peak ΔF/F magnitude, latency of the first evoked transient relative to the onset of the stimulus, rise time from the onset to the peak, half-width at half amplitude, and fall time from the peak to baseline. Results are shown for Hensen’s cells with ΔF/F > 1, Hensen’s cells with ΔF/F < 1, IPhCs, IBCs, clinocytes, clino2, and unnamed crista support cells. Significance of pairwise differences in means are provided in Tables 2–5 with “Y” indicating *p* < 0.05 (Fall time statistical significance is the same as half width). The two non-sensory cells with similarly large ΔF/F are Hensen’s cells and IBCs in the organ of Corti, and clino2 in the eminentia cruciatum. Although differences in peak ΔF/F exceeded an order of magnitude between cell types, differences between peak ΔF/F evoked by ATP vs. ACh within a cell type were small. There were, however, significant differences in onset latency and rise time between ACh and ATP in numerous cell types. These data suggest both stimuli are likely to trigger the same IP3 dependent CICR but, *via* unique receptor signaling pathways.

**TABLE 2:** ΔF/F: “Y” indicates difference was significant with *P* < 0.5.

			A	B	C	D	E	F	G	H	I	J	K	L	M	N
Crista support cell	ACh	A		Y	Y	Y	Y		Y		Y	Y		Y		Y
Clino2 cell	ACh	B	Y		Y											
Clinocyte	ACh	C	Y	Y												
Inner border cell (IBC)	ACh	D	Y				Y		Y		Y	Y		Y		Y
Inner phalangeal cell (IPhC)	ACh	E	Y			Y					Y	Y		Y		
Hensen’s cell A	ACh	F							Y	Y	Y	Y		Y		Y
Hensen’s cell B	ACh	G	Y			Y		Y			Y	Y		Y		
Crista support cell	ATP	H						Y			Y	Y		Y		Y
Clino2 cell	ATP	I	Y			Y	Y	Y	Y	Y						
Clinocyte	ATP	J	Y			Y	Y	Y	Y	Y						
Inner border cell (IBC)	ATP	K												Y		Y
Inner phalangeal cell (IPhC)	ATP	L	Y			Y	Y	Y	Y	Y			Y			
Hensen’s cell A	ATP	M														Y
Hensen’s cell B	ATP	N	Y			Y		Y		Y			Y		Y	

**TABLE 3:** Latency: “Y” indicates difference was significant with *P* < 0.5.

			A	B	C	D	E	F	G	H	I	J	K	L	M	N
Crista support cell	ACh	A					Y		Y			Y		Y	Y	Y
Clino2 cell	ACh	B														
Clinocyte	ACh	C														
IBC	ACh	D					Y	Y	Y	Y		Y	Y	Y	Y	Y
IPhC	ACh	E	Y			Y										
Hensen’s cell A	ACh	F				Y										
Hensen’s cell B	ACh	G	Y			Y										
Crista support cell	ATP	H				Y						Y		Y	Y	Y
Clino2 cell	ATP	I														
Clinocyte	ATP	J	Y			Y				Y						
IBC	ATP	K				Y								Y	Y	
IPhC	ATP	L	Y			Y				Y			Y			
Hensen’s cell A	ATP	M	Y			Y				Y			Y			
Hensen’s cell B	ATP	N	Y			Y				Y						

**TABLE 4:** Rise time: “Y” indicates difference was significant with *P* < 0.5.

			A	B	C	D	E	F	G	H	I	J	K	L	M	N
Crista support cell	ACh	A														
Clino2 cell	ACh	B								Y						
Clinocyte	ACh	C						Y		Y	Y				Y	
IBC	ACh	D						Y		Y	Y				Y	
IPhC	ACh	E								Y						
Hensen’s cell A	ACh	F			Y	Y										
Hensen’s cell B	ACh	G								Y	Y				Y	
Crista support cell	ATP	H		Y	Y	Y	Y		Y							
Clino2 cell	ATP	I			Y	Y			Y							
Clinocyte	ATP	J											Y	Y	Y	
IBC	ATP	K										Y			Y	
IPhC	ATP	L										Y				
Hensen’s cell A	ATP	M			Y	Y			Y			Y	Y			
Hensen’s cell B	ATP	N														

**TABLE 5:** Half width: “Y” indicates difference was significant with *P* < 0.5.

			A	B	C	D	E	F	G	H	I	J	K	L	M	N
Crista support cell	ACh	A														
Clino2 cell	ACh	B								Y						
Clinocyte	ACh	C				Y		Y		Y	Y			Y	Y	
IBC	ACh	D			Y			Y		Y	Y				Y	
IPhC	ACh	E						Y		Y	Y				Y	
Hensen’s cell A	ACh	F			Y	Y	Y									
Hensen’s cell B	ACh	G								Y	Y			Y	Y	
Crista support cell	ATP	H		Y	Y	Y	Y		Y							
Clino2 cell	ATP	I			Y	Y	Y		Y							
Clinocyte	ATP	J												Y	Y	
IBC	ATP	K												Y	Y	
IPhC	ATP	L			Y				Y			Y	Y			
Hensen’s cell A	ATP	M			Y	Y	Y		Y			Y	Y			
Hensen’s cell B	ATP	N														

### Gad2-Cre; PC-G5-tdT: Auditory Brainstem Response and Distortion Product Otoacoustic Emissions

**FIGURE 7:**
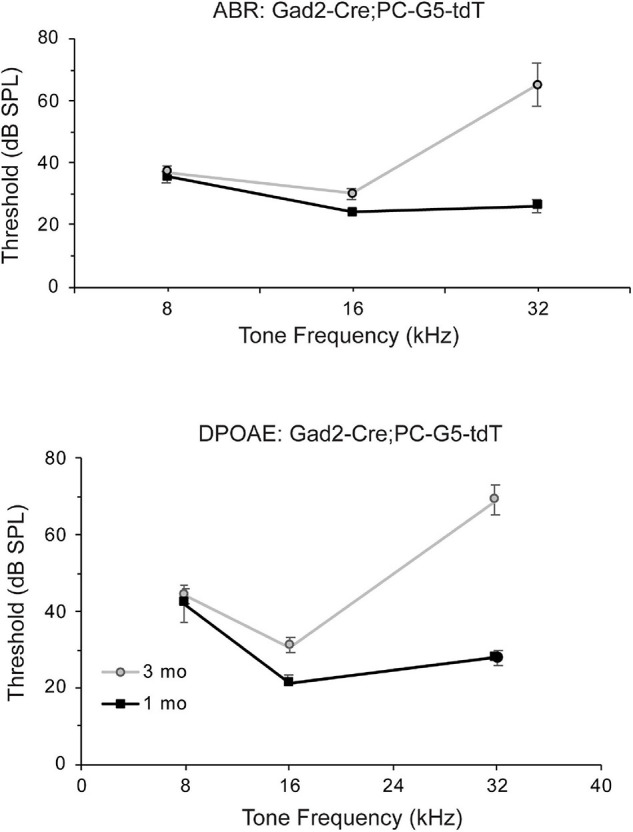
Auditory function of Gad2-Cre; PC-G5-tdT transgenic mice declines in young adults. Auditory function auditory brain stem (ABR) and distortion product otoacoustic emissions (DPOAEs) of Gad2-Cre; PC-G5-tdT were tested for two age groups [young (1 month; *n* = 5 mice) and adult (3 months; *n* = 5 mice)]. Young mice have normal thresholds for ABR and DPOAE tests. Adult mice have elevated amplitudes for ABR and DPOAE. Error bars represent mean ± SEM.

ABRs and DPOAEs were recorded in young and adult Gad2-Cre; PC-G5-tdT mice to show this hearing is unaffected by transgenic breeding. Auditory phenotypes were tested in two age groups; 1 and 3 months old male mice were tested at 8, 16, and 32 kHz. The ABR and DPOAE testing for 1 months old transgenic mice were within normal ranges (Figure 7). Threshold shifts for transgenic mice 3 months old were observed at 16 kHz and more pronounced at 32 kHz. These increases in thresholds are consistent with age relating hearing loss in the parental strains (C57BLKS/J, 129/SvEMS, C57BL/6J; Zheng et al., 1999).

## Discussion

Calcium transients in non-sensory and sensory cells play an essential role in the maturation of auditory and vestibular organs (Wang et al., 2015; Babola et al., 2018, 2020; Eckrich et al., 2018; Holman et al., 2020). In this study, we used acute cochlear vestibular explants from GCaMP5G expressing transgenic mice to record spontaneous, purinergic and cholinergic Ca^2+^ transients across sensory and non-sensory cells in the postnatal developing cochlea, semicircular crista and utricle. GCaMP5G is a relatively slow indicator most suitable for monitoring CICR and other changes in intracellular Ca^2+^ concentration lasting longer than 200 ms. This extends previous studies utilizing the Gad2 loci with a tdTomato-GCaMP5G reporter knock-in generating the Gad2-Cre; PC-G5-tdT transgenic mouse (Holman et al., 2019, 2020). Implications of a role for Gad2 in the sensory epithelia are not known at present and were not examined herein. However, the reproducible dual reporter expression from the Gad2 loci throughout sensory epithelia, especially in hair cells and support cells during development, provided a robust system to examine spontaneous and evoked Ca^2+^ transients throughout inner ear organs in a single animal.

In the neonatal mouse cochlea, ATP evoked Ca^2+^ transients in Hensen’s cells Deiters’ cells, IBCs, IPhCs, and IHCs were observed by postnatal day 4. Hensen’s cells responded with bursts of evoked Ca^2+^ transients following a puff application of ATP on postnatal days 5 and 6. Similar to other cells, we hypothesize that exogenous application of ATP activated purinergic receptors and triggered IP_3_ receptor–dependent CICR in non-sensory cells including Hensen’s and Deiters’ cells of the LER and IPhCs and IBCs of the GER. In vestibular organs in the same mice, ATP evoked Ca^2+^ transients primarily in supporting cells located in eminentia cruciatum (clinocytes, clino2) and two additional cell types labeled herein type A and B. The most intense ΔF/F Ca^2+^ transients observed in this study evoked by ATP occurred in the P6 utricle. These cells are most likely calyces, based on their morphology enveloping cells with type I hair cell shape. In the crista, clino2 cells responded with the highest intensity, and in the cochlea Hensen’s cells and IBCs responded with the highest intensity.

### Purinergic and Cholinergic Signaling in Neonatal Auditory and Vestibular Epithelia

In the mouse, spontaneous release of ATP occurs during cochlear development and ceases upon the maturation of hearing (Sugasawa et al., 1996; Lagostena and Mammano, 2001; Tritsch et al., 2007). Purinoceptors signaling regulates auditory neurotransmission (Housley et al., 1999; Järlebark et al., 2000; Lee and Marcus, 2008; Ito and Dulon, 2010), and contributes to tonotopy during development. ATP is likely released from IBCs through connexin hemichannels, thus modulating IHC activity (Zhao et al., 2005; Majumder et al., 2010; Tritsch et al., 2010; Dayaratne et al., 2014), while ACh released from olivocochlear efferent synaptic contacts might inhibit IHC activity (Glowatzki and Fuchs, 2000; Vetter et al., 2007; Maison et al., 2010; Johnson et al., 2011, 2013). An interplay between IHCs and IBCs is consistent with release of ATP by IBCs driving IHCs to release glutamate and excite spiral ganglion neurons during development (Zhao et al., 2005; Anselmi et al., 2008). Present results demonstrate large intracellular Ca^2+^ transients in response to ACh, revealing a potential mechanism to close the loop between spontaneous centripetal inputs to the CNS and cholinergic centrifugal feedback to the cochlea during development of tonotopy. Ca^2+^ transients in IBCs propagated between adjacent cells at a speed of approximately 2 μm-s^–1^, with the ΔF/F amplitude decreasing in the adjacent cell and decaying to zero after ∼3 cells. Results are consistent with intercellular communication *via* connexin gap junctions (Zhao et al., 2005; Anselmi et al., 2008; Zhu and Zhao, 2012; Dayaratne et al., 2014). In contrast to the cochlea, modest intercellular propagation of Ca^2+^ transients between adjacent VSCs indicate a similar developmental role might not be present in vestibular organs during the first postnatal week but it remains possible that a similar process takes place embryonically. Nevertheless, results suggest that supporting cell connexins might have a weaker role in vestibular epithelia relative to the cochlea during neonatal development. The fact that some connexin mutations cause severe hearing loss without vestibular impairment further supports this notion.

There are several limitations of the study worthy of note. First, results are comparative and observational in nature. Although previous work has established IP_3_ dependent CICR, as well as several cholinergic and purinergic receptors in the inner ear, the present study did not examine the molecular underpinnings of differences in Ca^2+^ transients or waves. Another limitation is the small sample size at each age, which prevents examination of precise timing of developmental changes occurring during the first postnatal week. Another consideration is the GCaMP5G transgenic line, which is derived from a parental strain (C57BL/6J) known to have age related hearing loss. A potential strength is the large number of cells and cell types with GCaMP5G examined within individual animals.

## Data Availability Statement

The raw data supporting the conclusions of this article will be made available by the authors, without undue reservation.

## Ethics Statement

The animal study was reviewed and approved by the University of Utah’s Institutional Animal Care and Use Committee.

## Author Contributions

HH and RR designed the study, conducted the experiments, analyzed the data, made the figures, and edited the manuscript. HH drafted the manuscript. Both authors contributed to the article and approved the submitted version.

## Conflict of Interest

The authors declare that the research was conducted in the absence of any commercial or financial relationships that could be construed as a potential conflict of interest.

## Publisher’s Note

All claims expressed in this article are solely those of the authors and do not necessarily represent those of their affiliated organizations, or those of the publisher, the editors and the reviewers. Any product that may be evaluated in this article, or claim that may be made by its manufacturer, is not guaranteed or endorsed by the publisher.
